# Red panda fine‐scale habitat selection along a Central Himalayan longitudinal gradient

**DOI:** 10.1002/ece3.5116

**Published:** 2019-04-01

**Authors:** Damber Bista, Prakash Kumar Paudel, Shant Raj Jnawali, Ang Phuri Sherpa, Saroj Shrestha, Krishna Prasad Acharya

**Affiliations:** ^1^ Red Panda Network Kathmandu Nepal; ^2^ Wildlife Science Unit, School of Agriculture and Food Sciences The University of Queensland Brisbane Queensland Australia; ^3^ Centre for Conservation Biology Kathmandu Institute of Applied Sciences Kathmandu Nepal; ^4^ WWF Nepal Kathmandu Nepal; ^5^ Ministry of Forests and Environment, Government of Nepal Singha Durbar Kathmandu Nepal

**Keywords:** *Ailurus fulgens*, bamboo, environmental variables, habitat selection, thermoregulation

## Abstract

Red panda *Ailurus fulgens*, an endangered habitat specialist, inhabits a narrow distribution range in bamboo abundance forests along mountain slopes in the Himalaya and Hengduan Mountains. However, their habitat use may be different in places with different longitudinal environmental gradients, climatic regimes, and microclimate. This study aimed to determine the habitat variables affecting red panda distribution across different longitudinal gradients through a multivariate analysis. We studied habitat selection patterns along the longitudinal gradient in Nepal's Himalaya which is grouped into the eastern, central, and western complexes. We collected data on red panda presence and habitat variables (e.g., tree richness, canopy cover, bamboo abundance, water availability, tree diameter, tree height) by surveys along transects throughout the species’ potential range. We used a multimodal inference approach with a generalized linear model to test the relative importance of environmental variables. Although the study showed that bamboo abundance had a major influence, habitat selection was different across longitudinal zones. Both canopy cover and species richness were unimportant in eastern Nepal, but their influence increased progressively toward the west. Conversely, tree height showed a decreasing influence on habitat selection from Eastern to Western Nepal. Red panda's habitat selection revealed in this study corresponds to the uneven distribution of vegetation assemblages and the dry climatic gradient along the eastern‐western Himalayas which could be related to a need to conserve energy and thermoregulate. This study has further highlighted the need of importance of bamboo conservation and site‐specific conservation planning to ensure long‐term red panda conservation.

## 1 INTRODUCTION

Understanding how a species selects its habitat and where and why it is distributed is a fundamental part of applied and theoretical research in animal ecology (Hull et al., 2014; Manly et al., 2002; Nielsen, McDermid, Stenhouse, & Boyce, 2010). It necessitates an understanding of dispersal mechanisms, behavioral plasticity, and habitat occupancy at fine‐spatial resolution across large landscapes (Hull et al., 2015; Kernohan, Gitzen, & Millspaugh, 2001; Powell, 2012). Studies of habitat occupancy based on coarse data such as weather stations and/or landscape features fail to account for fine‐scale habitat features, which may lead to erroneous conclusions about species distribution (Austin & Van Niel, 2011). Fine‐scale studies are important for gaining insight into extrinsic and intrinsic factors of species endangerment, particularly for endangered animals such as the red panda, *Ailurus fulgens* (Hull et al., 2014, 2015, Figure 1).

Species habitat occupancy is determined by a combination of environmental variables (Peterson, 2011). These include, among others, the bioclimatic envelope (e.g., temperature and precipitation), vegetation types, environmental structure, and topographic features (Jesse et al., 2017; Wiens, 2011). Although some variables may be correlated, there are species–specific responses, at different temporal and spatial scales, to environmental variables (Brown, 1999; Lawson, Bennie, Thomas, Hodgson, & Wilson, 2014).

Red pandas have a small longitudinal, latitudinal, and altitudinal distribution and inhabit a very narrow range of habitat and food requirements (Schaller, 1985; Wei, Feng, Wang, Zhou, & Hu, 1999; Yonzon & Hunter, 1991). They are taxonomically carnivores, but they have become specialized for herbivory, specifically bamboo (Roberts & Gittleman, 1984). Red pandas are now found in certain clusters within a narrow 2,100–4,325 m altitudinal range (Bista, Shrestha, Sherpa, et al., 2017; Dorjee, Chakraborty, & Dutta, 2014), mainly in montane forests (oak mixed, mixed broad‐leaf conifer, and conifer) with abundant bamboo in the understory (Glatston, Wei, Than Zaw, & Sherpa, 2015; Roberts & Gittleman, 1984). This species has been reported from Kalikot District of Nepal (81°E) in the west (Bista, Paudel, Ghimire, & Shrestha, 2016) to the Minshan Mountain and upper Min Valley in Sichuan Province, China (104°E) in the east (Choudhary, 1977; Ellerman & Morrision‐Scott, 1966).

Nepal, a central Himalayan country, has highly heterogeneous landscapes in terms of elevation and has one of the longest elevational gradients (70–8,848 m) within a short distance (Paudel & Heinen, 2015). The country is situated at the crossroads of the Paleotropic in the south and the Palearctic in the north (Udvardy, 1975), as well as at the confluence of several floristic regions (Welk, 2015). Much of the precipitation is brought by monsoons, which occur in a decreasing gradient from east to west. But winter rains, which account for nearly 20% of total precipitation, are common in western Nepal. As a consequence, Nepal has distinct microclimatic conditions and vegetation composition along a longitudinal gradient (Shrestha, Wake, Dibb, & Mayewski, 2000). Some of the significant variables may no longer be applicable as both climate and habitat characteristics change along gradients (Bearer et al., 2008; Hull et al., 2014; Kang, Yang, Li, Chen, & Zhao, 2013; Zhang et al., 2009).

This study aimed to analyze whether habitat selection by red pandas differs across various longitudinal complexes, in conjunction with climatic gradients. We used data on canopy cover, bamboo abundance, size of trees, water availability, tree height, and tree species richness collected throughout the habitat of red pandas in Nepal. The present work is pivotal for devising an appropriate conservation strategy for such an iconic member of the fauna of these mountain forests.

## 2 MATERIALS AND METHODS

### 2.1 Data collection

We collected data on the presence and absence of red panda and habitat variables (Table 1) along transects in its potential distribution range in Nepal (Figure 2). To develop a map of potential habitat of red panda, we examined 24 environmental variables (19 bioclimatic variables, four topographic variables, and one land cover variable). We obtained bioclimatic variables from WorldClim (http://www.worldclim.org) (version 1.4; Hijmans, Cameron, Parra, Jones, & Jarvis, 2005), topographic variables (elevation, aspect, and slope) from the website of the Shuttle Radar Topographic Mission (SRTM) (http://earthexplorer.usgs.gov), and land cover data from the webpage of the International Centre for Integrated Mountain Development were used (http://geoportal.icimod.org/). We developed a habitat suitability model using the Maximum Entropy Modeling in the MaxEnt software (MaxEnt version 3.3.3k) that correlates environmental variables with species occurrence using the maximum entropy principle (Phillips, Anderson, & Schapire, 2006). Presence data of red pandas were collected from various sources (Bhatta, Shah, Devkota, Paudel, & Panthi, 2014; Bista, Shrestha, Sherpa, et al., 2017; Kandel et al., 2015). Model performance was assessed based on area under the Receiver Operator Characteristic curve, known as AUC. The model with an AUC value of 0.92 was selected as potential habitat, which was used only for identification of sampling locations described in the following paragraph and therefore does not affect our results. We overlaid a grid map (9.6 km^2^) over the suitable habitat, where the size of each square was equivalent to the maximum home range of red panda as reported in Langtang National Park (Fox, Yonzon, & Podger, 1996; Yonzon, 1989). Grid cells with more than 50% area of suitable habitat were considered for random sampling. We randomly selected 50% of the suitable grid cells and overlaid each with 6 subgrids (area = 1.6 km^2^). Three of those subgrids were randomly sampled (MoFSC, 2016). In this way, a total of 557 subgrids were sampled across the entire habitat.

**Table 1:** List of variables collected during the survey

List of variables (abbreviation)	Description
Distance to water sources (disWat)	The shortest distance from the center of plot to the nearest water source (m)
Species composition (tree)	Number of tree species having diameter (DBH) ≤5 cm within the sampling plots
Tree diameter (DBH)	Diameter (cm) of all tree species measured at breast height above the ground (1.3 m) within a plot
Tree height (Treehigh)	Height of trees (m), all tree species located within the sampling plot (*A* = 314 m^2^), measured with the help of clinometer and visual estimation
Canopy cover (Canopy)	Canopy cover within the sampling plots measured by visual estimation (%)
Bamboo height	Height of bamboo, measured from the base to tip of each culm, falling within the subplot with radius 1 m (*A* = 3.14 m^2^) (m)
Number of bamboo culm	Total number of bamboo culms preset within the subplot (*A* = 1 m^2^) (number/m^2^)
Bamboo coverage	Bamboo cover within the subplot (*A* = 3.14 m^2^) based on visual estimation (%)

**Figure 1:**
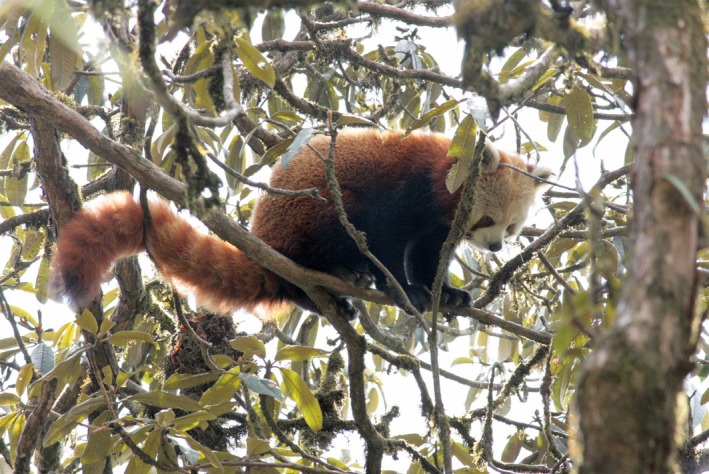
Red panda, an endangered species of the Himalaya

We placed three to five transects, each ranging from 500 to 1,000 m in length, along a 100 m contour within a subgrid. We walked slowly along each transect and recorded the presence or absence of red pandas in a concentric sampling plot with radius of 10 m (hereafter referred to as the sampling plot) at intervals of 500 m. We also recorded the presence data wherever presence signs were observed while traversing each transect, not limiting the observation to within these systematically established sampling plots. Presence was indicated by direct sighting or by observation of indirect signs such as droppings, footprints, foraging signs, scratch marks, or remains of a dead animal. We also recorded habitat variables in each plot (Table 1), which included number of tree species, tree diameter and height, and canopy cover in each plot (*A* = 314.28 m^2^). At the same time, we measured bamboo cover and bamboo height within a subplot of 1 m radius (*A* = 3.14 m^2^). We traversed a total of 1,451 km of transects and collected data from 2,935 plots, which included 590 plots with red panda presence signs.

### 2.2 Data analysis

**Figure 2:**
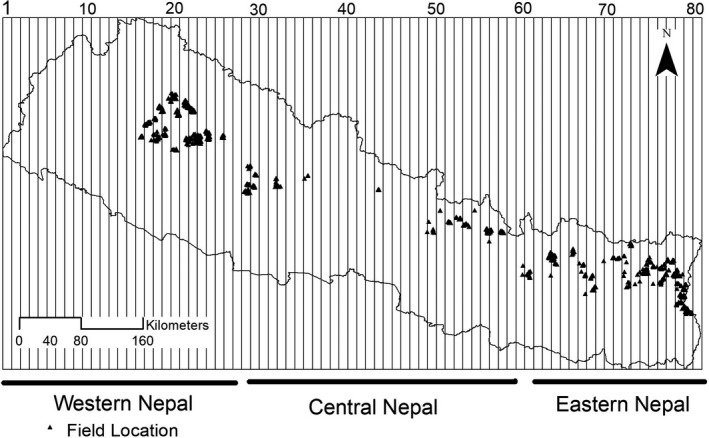
Map of Nepal showing longitudinal gradients. Entire Nepal Himalaya is divided into 80 1 km longitudinal gradient (one western most boundary, 80 eastern most boundary). The dots are sampled locations

Floristically, Nepal Himalaya is divided into three regions: western Nepal (west to 83°E in Nepal); central Nepal (83°E to 86°30'E); and eastern Nepal (east to 86°30'E) (Banerji, 1963; Press, Shrestha, & Sutton, 2000; Stainton, 1972; Figure 2). Hence, we stratified the entire country of Nepal into three longitudinal complexes corresponding with those floristic regions.

In order to assess the influence of habitat variables across three longitudinal complexes, we developed the following indices (Table 2) from data (Table 1) that describe habitat characteristics at plot levels:

**Table 2:** Description of habitat variables (indices) used in analyses (habitat variables distance to water, canopy cover are described in Table 1)

SN	Habitat variables (abbreviation)	Description
1	Tree species richness (SpeciesRichness)	We measured tree species diversity of each plot using a Menhinick's diversity index as $D=\frac{s}{\sqrt{N}}$; where *D* is Menhinick's diversity index, S is the number of species, and *N* is total abundance. The index reduces bias due to sampling effort and plot size (Whittaker 1977)
2	Bamboo abundance (BamboAbun)	Data on bamboo included number of bamboo plants, average bamboo height, and bamboo coverage (Table 1). We developed a simple index of bamboo abundance for each plot as *B* = *N* ^(log ^ *^H^* ^)^ × log (*c*); where, *B* is the bamboo abundance index, H is the average height of bamboo in a plot, and *c* is the bamboo coverage in a plot
5	Tree height (TreeHigh)	Average height of tree species within a sampling plot (see Table 1 for details)
6	Tree diameter (DBH)	Average DBH of all tree species in a sampling plot (see Table 1 for details)

We tested for multicollinearity among predictors, with the variance inflation factor (VIF) of each predictor using the USDM package (Naimi, 2017) in R (R Development Core Team, 2015). A VIF value >10 is regarded as severe multicollinearity (Montgomery & Peck, 1992). Our analysis suggested that all VIFs were lower than 2 suggesting that little or no collinearity among input variables (Table 3).

**Table 3:** Summary of variance inflation factor calculated from the results of multiple regression model

Variables	VIF
1. Distance to water	1.015
2. Tree height	1.029
3. Tree diameter	1.751
4. Canopy cover	1.219
5. Bamboo abundance	1.067
5. Tree species richness	1.588

We considered six habitat variables along longitudinal gradients (Table 2) and used a multi‐model inference approach to identify the subset of models with the most empirical support from a total of 64 possible logistic models (Burnham, Anderson, & SPRINGERLINK, 2002). We used Akaike's information criterion corrected for small samples (AICc) to rank the models. The models with AICc value >2 were considered to be better than competing models (Burnham et al., 2002).

We determined the importance of each habitat variable in a model by using model‐average parameter estimates (Calcagno, 2013). This is important because all variables were included in the same number of models (Doherty, White, & Burnham, 2012). We calculated parameter estimates of the best model using a generalized linear model (GLM) with binary response variables using glmulti package in R (R Development Core Team, 2015).

We obtained bioclimatic variables (maximum temperature of warmest months, precipitation of driest quarter, and temperature seasonality) from WorldClim (wordclim.org) and examined their gradients between 2,000 to 4,000 m elevation along longitudinal gradients (Figure 2) using Generalized Additive Model (GAM) in R (Supporting Information Appendices S1 and S2).

## 3 RESULTS

**Figure 3:**
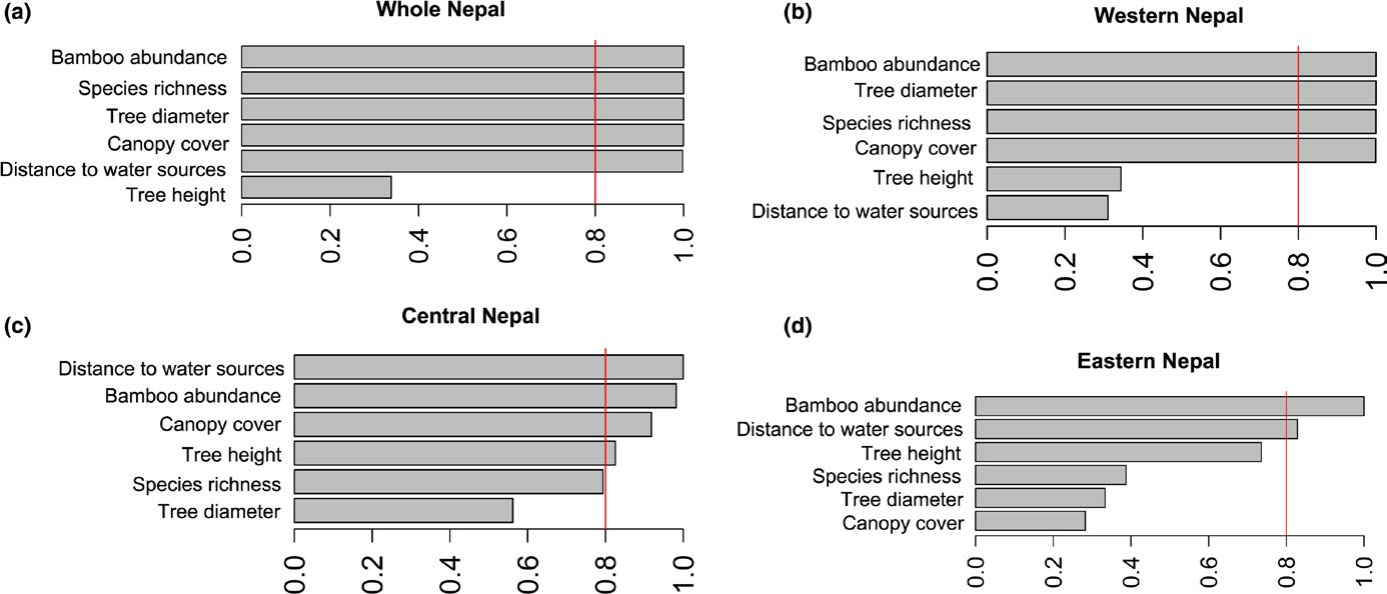
Model‐averaged importance of the habitat variables describing red panda occupancy in (a) whole Nepal, (b) western Nepal, (c) central Nepal, (d) eastern Nepal. The importance is based on the sum of Akaike weights derived from model selection using AICc (Akaike's information criterion corrected for small samples). Cutoff is set at 0.8 (dashed line) in order to differentiate among the most important predictors

Red panda distribution in Nepal was best explained by all variables except tree height (Table 4), with a strong preference for areas with high tree species richness, high bamboo abundance, close proximity to water sources, and high canopy cover; red pandas avoided sites with large tree diameter (Table 5, Figure 3a). Separate models for eastern, central, and western Nepal demonstrated different pattern of habitat selection. In eastern Nepal, red pandas showed a positive response to bamboo abundance and selected sites with taller tree and closer to water sources (Table 6). In the central Nepal, the best model included all variables (Table 4), where red pandas were positively associated with the canopy cover, tree species richness, and bamboo abundance, and negatively associated with the distance to water sources and tree height (Table 7). In the western Nepal, the best model for red panda occupancy included canopy cover, tree diameter, bamboo abundance, and tree species richness (Table 8). Here, red pandas significantly avoided sites with large tree diameters.

**Table 4:** Model selection results for GLMs comparing habitat variables for predicting red panda in Nepal Himalaya^a^

Model Specification			
SN	Model	AICc	weights
Nepal Himalaya			
1	PA ~ 1 + disWat + Canopy +DBH + BamboAbun +SpeciesRichness	1978.183	0.660
2	PA ~ 1 + disWat + Canopy +TreeHigh + DBH +BamboAbun + SpeciesRichness	1979.526	0.337
Eastern Nepal			
1	PA ~ 1 + disWat + TreeHigh + BamboAbun	857.236	0.171
2	PA ~ 1 + disWat + TreeHigh + BamboAbun + SpeciesRichness	857.873	0.124
3	PA ~ 1 + disWat + TreeHigh +DBH + BamboAbun	858.556	0.088
4	PA ~ 1 + disWat + Canopy +TreeHigh + BamboAbun	859.151	0.065
5	PA ~ 1 + disWat + BamboAbun	859.1759	0.064
Central Nepal			
1	PA ~ 1 + disWat + Canopy +TreeHigh + DBH + BamboAbun + SpeciesRichness	303.140	0.352
2	PA ~ 1 + disWat + Canopy + TreeHigh + BamboAbun +SpeciesRichness	304.143	0.213
Western Nepal			
1	PA ~ 1 + Canopy + DBH +BamboAbun + SpeciesRichness	747.482	0.450
2	PA ~ 1 + Canopy + TreeHigh +DBH + BamboAbun +SpeciesRichness	748.757	0.238
3	PA ~ 1 + disWat + Canopy + DBH + BamboAbun + SpeciesRichness	749.06	0.204

Abbreviation is defined in Table 1.

**Table 5:** Top GLM with binomial family measuring the influence of covariates on estimates of red panda occurrence in Nepal Himalaya

Parameters	Coefficient	*SE*	*z* value	*p* value
(Intercept)	−1.826	0.1569440	−11.641	<0.0001
DisWat	−0.001	0.0004683	−3.678	0.0002
Canopy	0.011	0.0027994	4.202	<0.0001
DBH	−0.001	0.0002678	−4.549	<0.0001
BamboAbun	0.129	0.0105073	12.354	<0.0001
SpeciesRichness	0.180	0.0291804	6.199	<0.0001

**Table 6:** Top GLM with binomial family measuring the influence of covariates on estimates of red panda occurrence in eastern Nepal

Parameters	Coefficient	*SE*	*z* value	*p* value
(Intercept)	−1.558	0.235	−6.609	<0.0001
disWat	−0.001	0.0007	−2.228	0.025
TreeHigh	0.026	0.013	1.983	0.047
BamboAbun	0.059	0.013	4.274	<0.0001

**Table 7:** Top GLM with binomial family measuring the influence of covariates on estimates of red panda occurrence in central Nepal

Parameters	Coefficient	*SE*	*z* value	*p* value
(Intercept)	−0.944	0.564	−1.672	0.09
disWat	−0.006	0.001	−4.968	<0.0001
Canopy	0.021	0.008	2.533	0.011
TreeHigh	−0.056	0.026	−2.170	0.03
DBH	−0.0009	0.0005	−1.697	0.089
BamboAbun	0.107	0.035	2.995	0.002
SpeciesRichness	0.205	0.094	2.178	0.029

**Table 8:** Top GLM with binomial family measuring the influence of covariates on estimates of red panda occurrence in western Nepal

Parameters	Coefficient	*SE*	z value	*p* value
(Intercept)	−2.219	0.246	−8.991	<0.0001
Canopy	0.018	0.004	4.133	<0.0001
DBH	−0.002	0.0004	−4.902	<0.0001
BamboAbun	0.192	0.018	10.289	<0.0001
SpeciesRichness	0.217	0.051	4.202	<0.0001

The model‐averaged importance of the habitat variables suggested bamboo abundance as the most important predictor in all longitudinal complexes (Figure 3). Canopy cover, tree diameter, and tree species richness were not important habitat variables in eastern Nepal, but they had increasing importance from central to western Nepal (Figure 3). Tree diameter was unimportant in eastern Nepal, but its importance increased in western Nepal.

## 4 DISCUSSION

Bamboo abundance was consistently a decisive factor of red panda occurrence in all longitudinal complexes in Nepal, corroborating similar studies (Bista, Shrestha, Sherpa, et al., 2017; Panthi, Khanal, Acharya, Aryal, & Srivathsa, 2017; Pradhan, Saha, & Khan, 2001; Wei et al., 1999; Yonzon & Hunter, 1991). There are marked variations in other predictors among longitudinal complexes. With the exception of western Nepal, red pandas favored sites closer to water sources. In western Nepal, canopy cover and tree diameter had greater influence, and red pandas preferred sites with high forest canopies and small tree diameters. Tree species richness was important and strongly favored in both central and western Nepal but was unimportant in eastern Nepal. These suggest habitat selection is determined by a set of interacting factors (Zhou et al., 2013) attributable to the different vegetation composition along the Himalayan longitudinal gradient (Shrestha et al., 2000).

The red panda diet is made up of 80% bamboo, and its availability is invariably important for red panda survival (Pradhan et al., 2001; Thapa, Hu, & Wei, 2018; Wei, Feng, Wang, & Hu, 2000; Wei et al., 1999; Williams, 2006; Yonzon & Hunter, 1991). Such a near‐inclusive reliance on bamboo may be a survival strategy. Bamboo is a low‐quality food (Hu, 2001; Schaller, 1985; Wei et al., 2000), and preference for such food lessens the travel needed to forage, which requires the expenditure of additional energy (Hu, 2001; Reid, Jinchu, & Yan, 1991). Additionally, red pandas may have adapted to utilize such fibrous low‐quality diets, not preferred by sympatric species, to avoid interspecies competition (Deueser & Shugart, 1978; Zhang, Wei, Li, & Hu, 2006). Some authors argue that habitats featuring medium levels of bamboo coverage are most suitable for red pandas, as high bamboo coverage increases travel time, requiring the expenditure of additional energy (Hu, 2001; Kang et al., 2013). Apart from bamboo, our study suggests that tree canopy coverage, proximity to water sources and species diversity were critical habitat requirements of red panda distribution, similar to the findings of previous studies (Bhatta et al., 2014; Bista, Shrestha, Sherpa, et al., 2017; Dorji, Vernes, & Rajaratnam, 2011; Pradhan et al., 2001; Thapa et al., 2018; Williams, 2006; Yonzon & Hunter, 1991). However, red pandas responded to these predictors differently in the three longitudinal complexes, suggesting diverse adaptations to physiological, thermoregulatory, and ecological constraints (Fei, Hou, Spotila, Paladino, & Zhang, 2017; McNab, 1989; Wang, 2015). Tree species richness, tree diameter, canopy cover, and tree height create unique climatic regimes at fine levels (Anhuf & Rollenbeck, 2001). We argue that selection of such habitat depends on bioclimatic factors that address these constraints.

Our results showed that tree canopy cover had no influence on red panda occurrence in eastern Nepal, but its influence increased progressively through central Nepal and western Nepal. Distance to water sources and tree species richness was more important predictors in eastern than western Nepal, but tree height showed a decreasing influence from eastern to western Nepal. We argue that such a pattern may be the result of longitudinal bioclimatic gradients that affect habitat selection of red pandas to conserve energy and thermoregulation. McNab (1989) has reported a decreased metabolic rate at low environmental temperatures without reducing body temperature in captive pandas. Red pandas should have low metabolic rates to conserve heat and thermoregulation, especially during winter (Fei et al., 2017; Wei et al., 2000), but they also need more foraging time to maximize energy intake from less nutritious bamboo (McNab, 1989; Yonzon & Hunter, 1991). Liu, Guan, Dai, Li, and Gong (2016) have reported temperature as the second most important determinant of giant panda distribution in the northern Minshan in China, similar to our findings.

In Nepal, rainfall is brought by the summer monsoon and winter rains. The summer monsoon accounts for about 80% of the precipitation (June–September) and is accompanied by a northwesterly airflow from the Bay of Bengal (Shrestha, 2000). The winter rain enters western Nepal via northern India and Kashmir (Lang & Barros, 2004). Thus, eastern Nepal is humid with short dry seasons. This may be why red pandas in this area occur in open‐canopy forests with tall trees and a bamboo understory. The tree canopy and stems reduce solar radiation penetration to near‐ground areas, and so below‐canopy microclimate regimes are usually humid (Geiger, Aron, & Todhunter, 1995; Hardwick et al., 2015), although forest structure, elevation, slope, aspect, and season have more influence (Ferrez, Davison, & Rebetez, 2011). The preference of red pandas for a more open‐canopy forest with taller tree in eastern compared to western Nepal could be the result of humid climatic conditions in such forests.

In eastern Nepal, red pandas preferred sites with close proximity to water sources and forests with tall trees. Such sites are perhaps important to conserve energy as they do not require long travel for water (Bista, Shrestha, Sherpa, et al., 2017; Pradhan et al., 2001). Tall tree branches may facilitate foraging for nutritious parts of bamboo, such as young leaves (Schaller, 1985; Wei & Zhang, 2011), and help red pandas avoid encounters with predators (Bista, Shrestha, Sherpa, et al., 2017; Dorji et al., 2011; Pradhan et al., 2001). In central Nepal, red pandas showed positive responses to canopy cover and tree species richness, along with closeness to water sources and shorter trees. While central Nepal has the lowest precipitation during dry months (Appendix 2), the tree canopy influences microclimates through attenuating and buffering variation in climatic conditions, that creates vertical gradients of mean temperature and humidity (Anhuf & Rollenbeck, 2001; Nakamura et al., 2017). This may provide optimal microclimatic conditions (e.g., temperature, relative humidity; Anhuf & Rollenbeck, 2001) and offer enhanced shelter and protection from predators (Pradhan et al., 2001; Yonzon & Hunter, 1991). Smaller trees seem to be crucial in providing a substrate for foraging on bamboo and probably preserve red panda energy by avoiding climbing tall trees. Bista, Shrestha, Sherpa, et al. (2017) reported a strong preference for a species‐rich forest (165 tree species) with abundant bamboo and water sources (<300 m) in central Nepal.

Our results showed an increasing preference for high tree canopy coverage and tree species diversity and a decreasing preference for large tree diameter from eastern to western Nepal. This is consistent with an increasing trend of maximum temperature of warm months along a longitudinal gradient (Appendix S1). Western Nepal receives late monsoon rain that spans a shorter period (Shrestha, 2000). This creates an east to west precipitation gradient, resulting in a drier environment in western Nepal (Shrestha et al., 2000), although western Nepal receives more winter rain (Kansakar, Hannah, Gerrard, & Rees, 2004; Shrestha, 2000), and seasonal variation in temperature increases moving west (Appendix 2). As a consequence, red pandas seem to prefer closed forests dominated by dwarf tree species that provide better protection from dryness and cold temperatures, allowing them attain their optimum energy expenditure. Mammals have a thermal neutral zone in which animals have a minimal metabolic rate (Withers, 1992), and red pandas probably try to maintain their thermoregulation by living in a habitat within that temperature range (Fei et al., 2017). Preference for such forests here may be related to the optimal temperature created in the forest understory. Western Himalaya has high bamboo abundance in comparison with the other two zones, but the height of the bamboo culms constitutes a limiting factor for them to determine what species to feed on. Higher the bamboo stems are, the lower the availability of bamboo leaves for animals (Wei & Zhang, 2011). Trees with large diameters are taller and more difficult for red pandas to climb. This could be a reason for avoiding sites with tall trees with large diameters, as those trees are unlikely to serve as substrate. Feeding on leaves and fruits of other tree species have been well documented in western Nepal (Panthi, Coogan, Aryal, & Raubenheimer, 2015; Sharma, Swenson, & Belant, 2014). Preference for sites with diverse vegetation could be a survival strategy to remain resilient in unfavorable situations. Interestingly, distance to water sources was found insignificant in western Nepal, which could be attributed to the availability of water in small ditches and tree holes, which may last a long time in closed canopy forests. However, further study is necessary to shed light on these aspects.

### 4.1 Implications for red panda conservation

Our results unveil fine‐scale habitat selection pattern of red panda in Nepal Himalaya. The bioclimatic gradients created by precipitation pattern seem to be the prime factor behind this uneven vegetation composition and associated microhabitat variation. This study has further highlighted the need for site‐specific conservation strategies rather than relying on a general plan for the entire habitat range in Nepal (Acharya et al., 2018). In general, availability of bamboo seems to be the primary predictor of red panda distribution in all three longitudinal zones. Therefore, bamboo conservation should be the topmost priority to ensure red panda survival in the long run. Any future conservation plan for red panda needs to be focused on creating species‐rich, dense forests in western Nepal. This is particularly important here as most red panda habitats are situated outside of protected areas (Bista, Shrestha, Kunwar, et al., 2017; Bista, Shrestha, Sherpa, et al., 2017; Thapa et al., 2018), and silviculture practices promoting selected tree species in community forests may further jeopardize survival of this species.

Many bamboo species are vulnerable to climate change because of their unusual extended reproduction intervals, ranging from 10 to 120 years (Janzen, 1976), along with limited seed dispersal ability (Mao‐Ning et al., 2012; Taylor, Reid, Zisheng, & Jinchu, 1991). The giant panda's habitat in China has been predicted to shift toward higher elevations, resulting in a decline of existing habitat by 60% within the next 70 years (Songer, Delion, Biggs, & Huang, 2012). Loss of bamboo could result in habitat fragmentation, making most of the current habitat unsuitable in the future. Therefore, creation of red panda‐focused conservation zones is needed to secure the long‐term survival of red pandas through maintaining habitat connectivity in Nepal. Establishment of corridors to facilitate the movement of red pandas during unfavorable situations (eg. forest fires and bamboo loss during a mass die‐off) is equally important. Additionally, red pandas’ positive response to higher tree species diversity indicates the need for conservation of other important tree species that provide shelter for resting, nesting, and supplementary diet. Likewise, conservation of water sources should be duly prioritized at watershed levels for enriching habitat quality.

Red pandas selected habitats differently in different bioclimatic conditions, although bamboo is the primary predictor of red panda distribution. This study suggests that the red panda is more of a habitat specialist than previously believed. Therefore, this study suggests a need for site‐specific conservation planning which could also be applicable for other specialist species. Such a plan needs to be bolstered with detailed information of bamboo distribution, presence of other palatable species, and microclimatic conditions. Moreover, climatic change‐induced range shifts may have profound impacts on the prospect of future survival. Scientific research on such issues is very few and therefore urgently needed. This is why we also recommend study on thermoregulatory adaptation of red pandas in the wild during the summer and winter seasons.

## CONFLICT OF INTEREST

The authors declare that they have no conflict of interest.

## AUTHOR CONTRIBUTIONS

DB, SRJ, APS, and KPA conceived the idea. DB and PKP designed the methodology. DB and SS collected the data. DB and PKP contributed to statistical analysis. DB and PKP prepared the draft of the manuscript. SRJ, APS, KPA, and SS provided inputs for revision and all coauthors approved the manuscript.

## Data Availability

Data have been archived in Dryad with https://doi.org/10.5061/dryad.77r7b60.
