# Supplementary material for: Red panda fine‐scale habitat selection along a Central Himalayan longitudinal gradient
Source: Ecol Evol. 2019 Apr 1;9(9):5260–9. doi: 10.1002/ece3.5116 (PMC6509368; doi:10.1002/ece3.5116)
Supplement: Supplementary file 1 [file ECE3-9-5260-s001.docx]

# **Appendix**


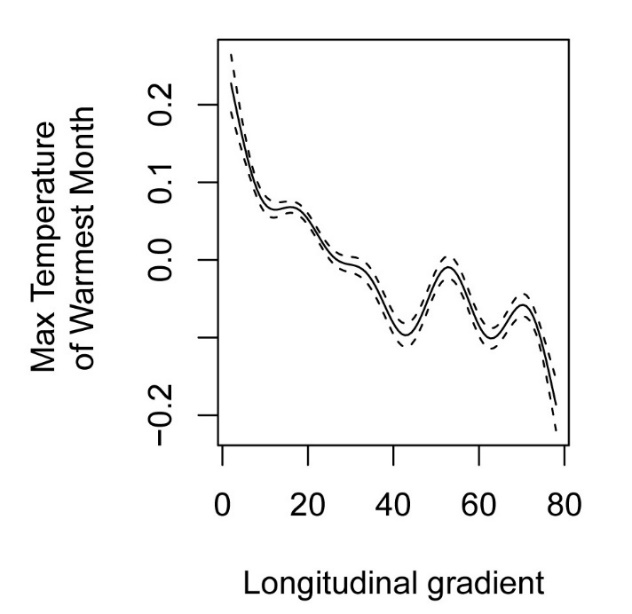


Appendix 1. Maximum temperature of warmest months along longitudinal gradient. Entire Nepal Himalaya is divided into 80 1 km longitudinal gradient (1 western most boundary, 80 eastern most boundaries).


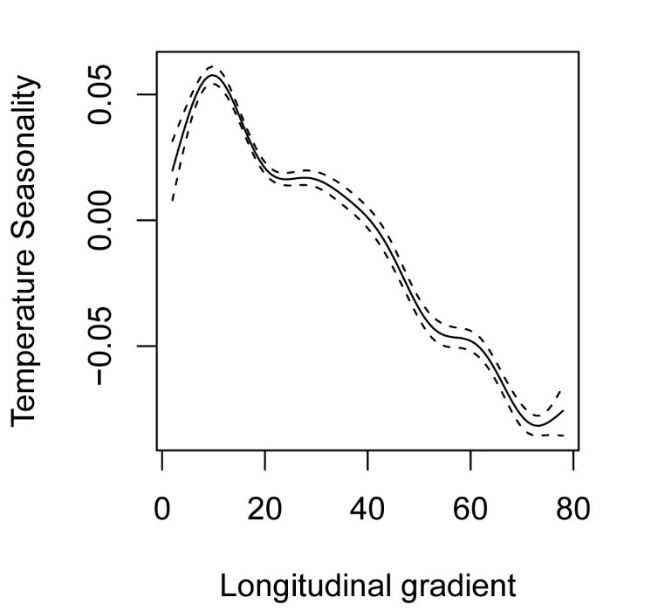


Appendix 2. Temperature seasonality along longitudinal gradient. Entire Nepal Himalaya is divided into 80 1 km longitudinal gradient (1 western most boundary, 80 eastern most boundaries).
